# Highly Expression of CD11b and CD32 on Peripheral Blood Mononuclear Cells from Patients with Adult-Onset Still’s Disease

**DOI:** 10.3390/ijms18010202

**Published:** 2017-01-19

**Authors:** Hyoun-Ah Kim, Bunsoon Choi, Chang-Hee Suh, Mi Hwa Han, Ju-Yang Jung, Hasan M. Sayeed, Ye Won Kim, Seonghyang Sohn

**Affiliations:** 1 Department of Rheumatology, Ajou University School of Medicine, 164 Worldcup-ro, Yeongtong-gu, Suwon 443-380, Korea; chsuh@ajou.ac.kr (C.-H.S.); clearmifa@hanmail.net (M.H.H.); serinne20@hanmail.net (J.-Y.J.); Kyw1016@hanmail.net (Y.W.K.); 2 Department of Microbiology, Ajou University School of Medicine, 164 Worldcup-ro, Yeongtong-gu, Suwon 443-380, Korea; blueppang@aumc.ac.kr; 3 Department of Biomedical Science, Ajou University School of Medicine, 164 Worldcup-ro, Yeongtong-gu, Suwon 443-380, Korea; Sayeed@ajou.ac.kr

**Keywords:** adult-onset still’s disease, pattern recognition receptor, CD11b, CD32, disease activity

## Abstract

Background: We investigated the potential role of several pattern-recognition receptors (PRRs; CD11b, CD11c, CD32, CD206, CD209, and dectin-1) in adult-onset Still’s disease (AOSD). Methods: The study included 13 untreated AOSD patients, 19 rheumatoid arthritis (RA) patients (as a disease control), and 19 healthy controls (HCs). The PRRs were quantified in peripheral blood using flow cytometry. The serum levels of interleukin-17 (IL-17), IL-18, and IL-23 were measured by enzyme-linked immunosorbent assay. Results: Significantly higher mean frequencies of cells presenting CD11b and CD32 from whole blood were observed in patients with AOSD than in patients with RA or HC. The levels of IL-17, IL-18, and IL-23 were elevated in AOSD patients compared to HCs. CD11b frequencies from whole cells correlated with systemic scores, lactate dehydrogenase (LDH) levels, aspartate transaminase levels, interleukin-23 (IL-23) levels, and IL-18. Frequencies of CD209 from granulocytes were significantly correlated with systemic scores, and the erythrocyte sedimentation rate and levels of C-reactive protein, ferritin, LDH, IL-23, and interleukin-18 (IL-18). Conclusions: Elevated frequencies of circulating CD11b-positive cells and positive correlations with disease activity markers suggest that circulating CD11b-positive cells contribute to the pathogenesis of AOSD.

## 1. Introduction

Adult-onset Still’s disease (AOSD) is an uncommon systemic inflammatory disease that is also known as the adult form of systemic juvenile idiopathic arthritis (JIA) [1]. It is characterized by high spiking fever, an evanescent salmon color maculopapular eruption, arthritis, and sore throat. Although the pathogenesis of AOSD is unclear, several factors, including a predisposing genetic background, environmental factors, and immune dysregulation have been suggested to contribute to the development of the disease [1,2]. Some studies have reported an association between AOSD and human leukocyte antigen (HLA) alleles, such as HLA Bw35 and HLA-DRB1, but the data remain inconclusive [1,3,4]. Several clinical features of AOSD are similar to infections, such as fever, rash, and lymphadenopathy, suggesting infection may be implicated in the pathogenesis of AOSD [5]. Viral or bacterial infections have been reported as a possible cause or trigger of AOSD [1,2,5,6,7]. Also, a role for proinflammatory cytokines, including interleukin-1β (IL-1β), IL-6, and IL-18, has been suggested in the pathogenesis of AOSD [8,9,10].

Pattern recognition receptors (PRR) play critical roles in pathogen recognition to initiate immune responses that ultimately link to the generation of adaptive immunity [11]. Also, specific PRRs detect host-derived damage-associated molecular patterns (DAMPs). Many families of proteins have been identified or confirmed as PRRs, the activation of which leads to the induction of proinflammatory cytokines. Thus, many PRRs have been suggested to be linked to numerous inflammatory and infectious diseases. In particular, several studies have revealed a key role of Toll-like receptors (TLRs) in host immune responses among PRRs [12]. S100A8/A9 is known as an endogenous TLR4 ligand, and has been reported to form a positive feedback loop with IL-1β in systemic JIA [13]. We also showed that S100A8/A9 levels were elevated in AOSD patients, and S100A8/A9 levels correlated with disease activity markers in AOSD [14]. Thus, PRRs and DAMPs, including S100A8/A9, could contribute to the pathogenesis of AOSD, and connect viral and/or bacterial infections to immune dysfunction.

Many PRRs have been reported in several chronic inflammatory diseases. Plasma mannose-binding lectin was significantly high in patients with systemic lupus erythematosus (SLE) compared with healthy control (HC), and correlated with disease activity markers [15]. The level of CD209 was higher in psoriasis vulgaris lesions than normal tissues [16]. Also, one report showed that monocytes from SLE patients showed diminished expression of dectin-1 compared with HC [17]. However, there are few data concerning any association of PRRs with disease activity in AOSD patients.

This study investigated the potential role of several PRRs (CD11b, CD11c, CD32, CD206, CD209, and dectin-1) in the pathogenesis of AOSD. These markers were quantified in peripheral blood from AOSD patients, rheumatoid arthritis (RA) patients, and HC using flow cytometry. The correlations between the frequencies of several PRRs and disease activity were investigated in AOSD patients.

## 2. Results

### 2.1. Clinical Characteristics of the Patients

Table 1 summarizes the clinical characteristics of the 13 patients with AOSD, the 19 with RA, and the 19 HC. The mean age of the AOSD patients was 51.1 ± 20.5 years and females comprised 84.6% of all patients. There was no significant difference in age or gender among the AOSD patients, the RA patients, and the HC. The AOSD patients manifested arthritis (*n* = 11, 84.6%), a high spiking fever (*n* = 12, 92.3%), skin rash (*n* = 9, 69.2%), and a sore throat (*n* = 9, 69.2%). Of the 13 AOSD patients, eight were in the process of experiencing their first event. The remaining five patients experienced a systemic flare-up of the disease during follow-up, and had discontinued their medication before the flare-up. Among eight patients exhibiting high-level disease activity prior to treatment, five patients had AOSD of the monophasic pattern, and three had disease of the chronic articular pattern. The AOSD patients did not have any hemophagocytic features at the time of sampling. Also, follow-up samples were collected from five patients after resolution of disease activity at 4 ± 1 months after the first samplings.

### 2.2. Percentage of Surface-Stained Cells Presenting CD11b, CD11c, CD32, CD206, CD209, and Dectin-1 in AOSD Patients, RA Patients, and HC

**Figure 1:**
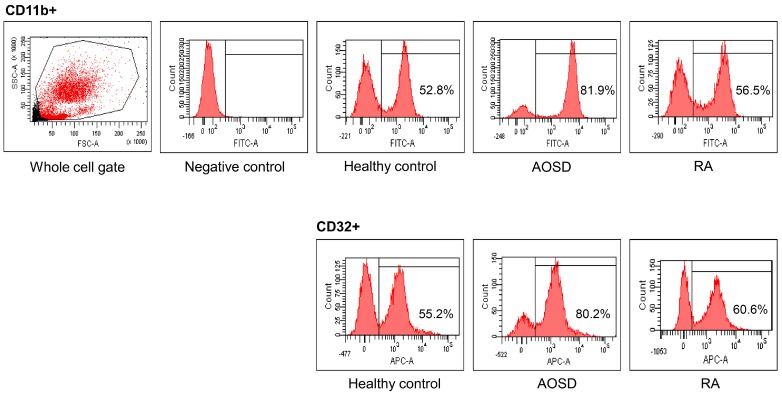
Representative examples of flow cytometric histograms of surface-stained cells presenting several cellular markers from peripheral blood of one patient with adult-onset Still’s disease (AOSD), a patient with rheumatoid arthritis (RA), and a healthy control (HC) are shown. PBMCs were treated with ACK solution to lyse red blood cells (RBCs) and washed with phosphate-buffered saline (PBS), after which 1 × 10^6^ cells in each tube were incubated with allophycocyanin (APC)-labeled anti-CD4, fluorescein isothiocyanate (FITC)-labeled anti-CD11b, and APC-labeled anti-CD32, for 30 min at 4 °C. The stained cells were then washed with PBS and analyzed using a flow cytometer (FACSAria III; Becton Dickinson, San Jose, CA, USA) with 10,000 cells.

**Figure 2:**
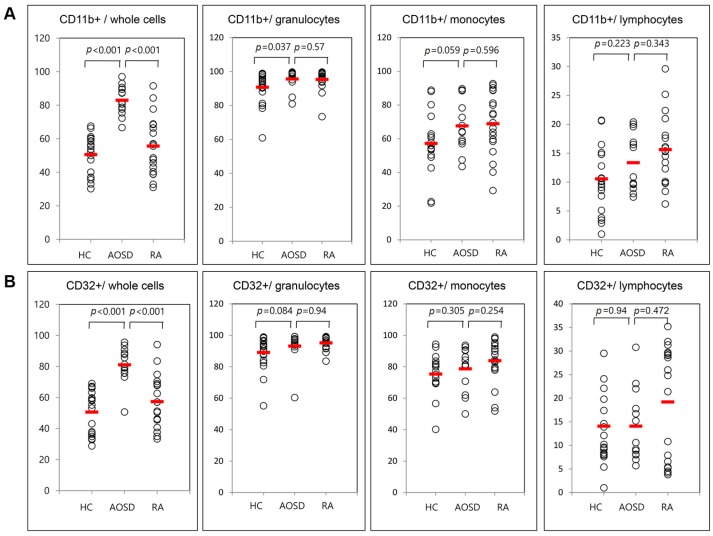
Flow cytometric results of the percentage of surface-stained cells presenting CD11b (**A**); and CD32 (**B**) in patients with adult-onset Still’s disease (AOSD), patients with rheumatoid arthritis (RA) and healthy controls (HC). Results were obtained from 13 patients with AOSD, 19 RA patients, and 19 HC. The horizontal line indicates the mean value for each group. The *p*-value was determined with the Mann–Whitney *U*-test.

Representative examples of flow cytometric histograms of surface-stained cells presenting several cellular markers from PB of one patient with AOSD, a patient with RA, and a HC are shown (Figure 1). Significantly higher mean frequencies of surface-stained cells presenting CD11b from whole blood were observed in patients with AOSD (82.9% ± 8.8%) than in patients with RA (55.6% ± 17.3%, *p* < 0.001) or in HC (50.5% ± 11.7%, *p* < 0.001; Figure 2A). Significantly higher frequencies of cells presenting CD32 from whole blood were seen in patients with AOSD (80.3% ± 11.4%) than in patients with RA (57.4% ± 16.9%, *p* < 0.001) or HC (50.6% ± 14%, *p* < 0.001; Figure 2B). However, there was no significant difference in the percentage of cells presenting CD11c, CD206, CD209, or dectin-1 between AOSD and RA, or between AOSD and HC.

### 2.3. Serum Levels of IL-17, IL-18, and IL-23 in AOSD Patients, RA Patients, and HC

**Figure 3:**
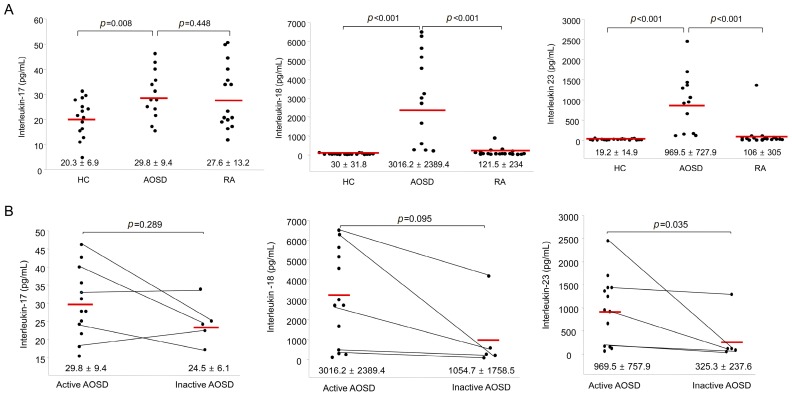
(**A**) Levels of serum interleukin-17 (IL-17), IL-18, and IL-23 in AOSD patients, RA patients, and healthy controls (HC). The horizontal line indicates the mean value for each group. The *p*-value was determined with the Mann–Whitney *U*-test; (**B**) Levels of serum IL-17, IL-18, and IL-23 according to disease activity in patients with AOSD. The horizontal line indicates the mean value for each group. The *p*-value was determined with the Mann–Whitney *U*-test.

Figure 3A shows the IL-17, IL-18, and IL-23 levels in AOSD patients, RA patients and HC. The IL-17 levels in AOSD patients (29.8 ± 9.4 pg/mL) were higher than those in HC (20.3 ± 6.9 pg/mL, *p* = 0.009). However, there was no significant difference in the levels of IL-17 between AOSD patients and RA patients. The IL-18 levels of AOSD patients (3016.2 ± 2389.4 pg/mL) were higher than those of RA patients (121.5 ± 234 pg/mL, *p* < 0.001) and HC (30 ± 31.8 pg/mL, *p* < 0.001). Also, the IL-23 levels of AOSD patients (969.5 ± 727.9 pg/mL) were higher than those of RA patients (106 ± 305 pg/mL, *p* < 0.001) and HC (19.2 ± 14.9 pg/mL, *p* < 0.001).

The levels of IL-17 and IL-18 were not different between active and inactive phases. Significantly higher IL-23 levels were observed in patients with active AOSD (969.5 ± 757.9 pg/mL) than in patients with inactive AOSD (325.3 ± 237.6 pg/mL, *p* = 0.035; Figure 3B).

### 2.4. Percentages of Surface-Stained Cells Presenting CD11b, CD11c, CD32, CD206, CD209, and Dectin-1, and Serum Levels of IL-17, IL-18, and IL-23 According to Disease Activity in AOSD Patients

**Figure 4:**
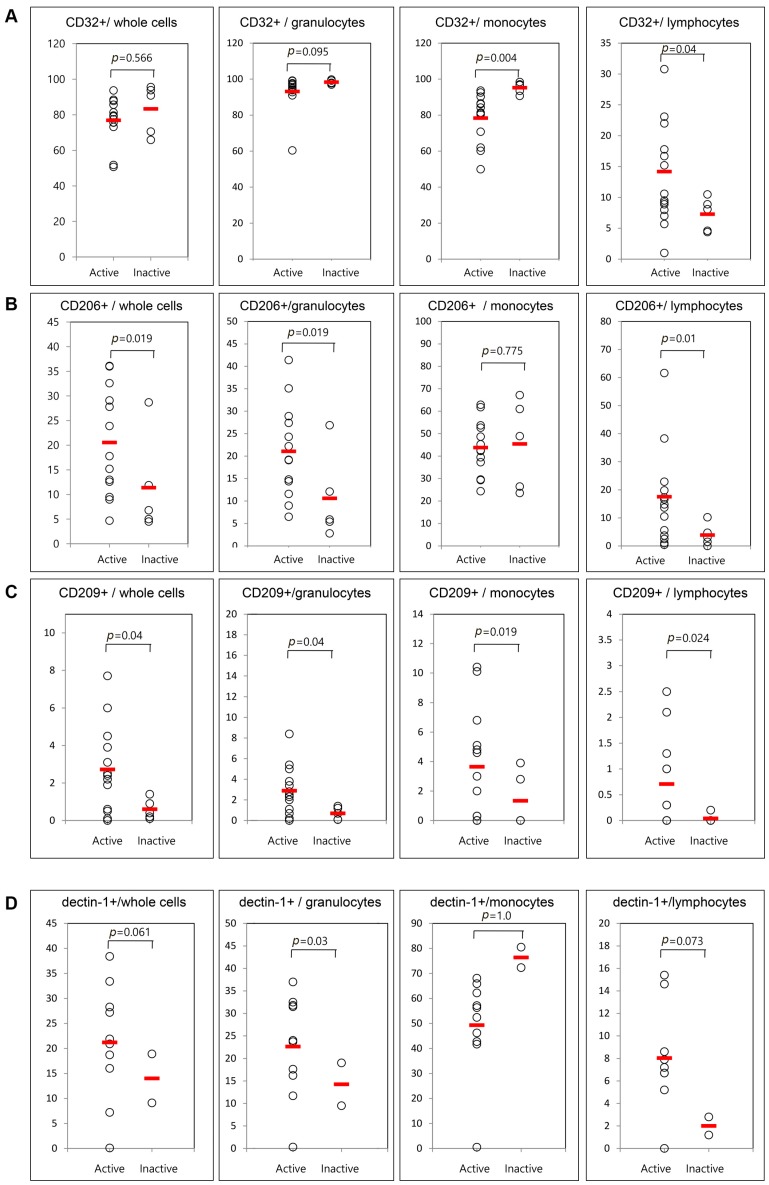
Flow cytometric results of the percentage of surface-stained cells presenting CD32 (**A**); CD206 (**B**); CD209 (**C**); and dectin-1 (**D**) according to disease activity in patients with AOSD. The results were obtained from 18 samples from AOSD patients. Thirteen active, untreated AOSD patients were included, and follow-up samples were collected from five patients after their disease activity resolved. The horizontal line indicates the mean value for each group. The *p*-value was determined with the Mann–Whitney *U*-test.

Only five patients were available for examination in both the active and inactive phases. Therefore, we compared the frequencies of cells presenting CD11b, CD11c, CD32, CD206, CD209, and dectin-1 between 13 active AOSD patients and five inactive AOSD patients using follow-up data (Figure 4). The frequencies of circulating CD11b and CD11c were not different between active and inactive phases. Significantly lower mean frequencies of cells presenting CD32 from monocytes were observed in patients with active AOSD (78.7% ± 13.9%) than in patients with inactive AOSD (94.4% ± 4.8%, *p* = 0.004). Also, significantly higher frequencies of cells presenting CD32 from lymphocytes were observed in patients with active AOSD (14.1% ± 7.6%) than in those with inactive AOSD (7.6% ± 2.9%, *p* = 0.04). The frequencies of CD206 from whole blood cells were increased in patients with active AOSD (21.1% ± 10.2%) versus inactive AOSD (9.9% ± 10.5%, *p* = 0.019). The frequencies of CD206 from granulocytes and lymphocytes were increased in patients with active AOSD versus inactive AOSD (*p* = 0.019 and 0.01, respectively). The frequencies of CD209 from whole blood cells were increased in active AOSD patients (2.8% ± 2.2%) versus inactive AOSD patients (0.3% ± 0.4%, *p* = 0.04). The frequencies of CD209 from granulocytes, monocytes, and lymphocytes were increased in active AOSD patients versus inactive AOSD patients (*p* = 0.04, 0.019, and 0.024, respectively). The frequencies of dectin-1 from granulocytes were increased in patients with active AOSD (24.5% ± 8.4%) versus those inactive AOSD (4.9% ± 6.5%, *p* = 0.03).

### 2.5. Correlation between the Frequencies of Stained Cells Presenting Several Markers and Disease Activity Markers in AOSD Patients

Correlations between the levels of disease activity markers and the frequencies of stained cells presenting several markers in AOSD patients are shown in Table 2. CD11b frequencies from whole cells correlated with systemic scores (*r* = 0.487, *p* = 0.041), and levels of lactate dehydrogenase (LDH) (*r* = 0.58, *p* = 0.012), aspartate transaminase (AST) (*r* = 0.635, *p* = 0.005), IL-23 (*r* = 0.529, *p* = 0.024), and IL-18 (*r* = 0.529, *p* = 0.015). The frequencies of CD11c from lymphocytes correlated with ESR (*r* = 0.541, *p* = 0.02), ferritin (*r* = 0.672, *p* = 0.002), and LDH (*r* = 0.539, *p* = 0.021). There was no correlation between CD32 from whole cells and the levels of disease activity markers. However, the frequencies of CD32 from monocytes were negatively correlated with systemic score (*r* = −0.559, *p* = 0.016), CRP (*r* = −0.468, *p* = 0.05), ferritin (*r* = −0.583, *p* = 0.011), and IL-23 (*r* = −0.476, *p* = 0.046). The frequencies of CD209 from whole cells were correlated with systemic score (*r* = 0.486, *p* = 0.041) and ferritin (*r* = 0.688, *p* = 0.002). Furthermore, the frequencies of CD209 from granulocytes correlated positively with systemic scores (*r* = 0.484, *p* = 0.021), and the levels of ESR (*r* = 0.452, *p* = 0.03), CRP (*r* = 0.428, *p* = 0.038), ferritin (*r* = 0.705, *p* = 0.001), LDH (*r* = 0.535, *p* = 0.011), IL-23 (*r* = 0.458, *p* = 0.028), and IL-18 (*r* = 0.411, *p* = 0.045). The frequencies of dectin-1 from lymphocytes correlated with ferritin (*r* = 0.829, *p* = 0.001), LDH (*r* = 0.694, *p* = 0.009), and IL-23 (*r* = 0.525, *p* = 0.049).

We evaluated the correlations between each cell surface marker in the patients with AOSD. The frequencies of CD11b from whole cells correlated with the frequencies of CD32 from whole cells (*r* = 0.853, *p* < 0.001). The frequencies of CD11c correlated with the frequencies of CD206 (*r* = 0.765, *p* < 0.001) and those of dectin-1 (*r* = 0.811, *p* = 0.001). The frequencies of CD206 were positively correlated with CD209 (*r* = 0.624, *p* = 0.003) and dectin-1 (*r* = 0.846, *p* < 0.001). Also, the frequencies of dectin-1 correlated with those of CD209 (*r* = 0.615, *p* = 0.017) (Table 3).

## 3. Discussion

This study showed significantly higher frequencies of cells presenting CD11b and CD32 from whole blood cells in patients with AOSD than in patients with RA or in HC. The CD11b frequencies from whole blood cells correlated with several disease activity markers, such as systemic score, LDH, AST, IL-23, and IL-18 levels. Furthermore, the frequencies of CD209 from granulocytes correlated positively with systemic scores, and the levels of ESR, CRP, ferritin, LDH, IL-23, and IL-18.

CD11b is a complement receptor 3 molecule expressed by phagocytes, such as neutrophils and monocytes; CD11b is also known as a prominent PRR that recognizes DAMPs and pathogen-associated molecular patterns (PAMPs), including HMGB1, DNA, and β-glucan [18,19,20]. Also, CD11b/CD18 (Mac-1) is a β2 integrin known as a pro-inflammatory molecule that promotes phagocyte cytotoxic function and enhances the function of several effector molecules [21]. A recent study showed that CD11b expression in neutrophils and monocytes was elevated in severe sepsis versus non-infectious conditions [22]. However, some studies have reported that Mac-1 plays significant immunoregulatory roles, and variations of the ITGAM gene, which encodes the CD11b chain, are a strong genetic risk factor in SLE [21,23]. CD32 is the most widely expressed of the FcγR, and is found on monocytes, neutrophils, B cells, and NK cells [24]. There are three different isoforms, FcγRIIA, FcγRIIB, and FcγRIIC, and they have different cellular expression, function, and ligand binding specificity [25]. In particular, FcγRIIA is predominantly expressed on monocytes and neutrophils, and signals the cell to increase phagocytosis and to secrete cytokines when it meets immunoglobulin G (IgG) [26]. In autoimmune diseases, one study showed that the expression of CD32 was reduced on peripheral blood monocytes from patients with SLE [27]. However, increased CD32 expression has been reported on peripheral blood monocytes from patients with RA [28,29]. In our study, leukocyte CD11b expression was significantly increased in patients with AOSD versus patients with RA or HC. Furthermore, leukocyte CD11b frequencies correlated significantly with levels of several disease activity markers and proinflammatory cytokines, such as IL-23 and IL-18. Also, leukocyte CD32 expression was significantly increased in patients with AOSD compared with patients with RA or HC. These results suggest that CD11b leukocytes may play a role in the pathogenesis of AOSD and clinical manifestations, and may serve as a marker for the evaluation of disease activity. However, the frequencies of circulating CD11b and CD32 were not different between the active and inactive phases of AOSD. It is not clear why this marker is elevated continuously in inactive AOSD patients. However, in the previous study, the inactive phase of systemic JIA on medication was defined as having no clinical symptoms observed in the active phases, as well as normal ESR and CRP levels, similar to our inactive AOSD patients [30]. And the remission was defined as the six continuous months of inactive disease while on medication. One study reported that serum cytokine (soluble ST2) levels in patients with systemic JIA were elevated even in the inactive phase, although other clinical parameters were normalized [31]. And they suggested that the result was related that certain of their follow up patients were in the inactive phase, not remission condition, similar to the previous definition for systemic JIA. Our current results could be similar to those conditions. In this study, follow-up samples were collected from five patients with inactive phase at 4 ± 1 months after the first samplings. In effect, two patients had flare after the second sampling. Also, the number of follow-up samples was small for subgroup analysis. Therefore, further work with larger sample sizes is required to evaluate the effects of CD11b in the pathogenesis of AOSD. Interestingly, CD32 frequencies of monocytes were increased significantly in inactive versus active AOSD. However, CD32-positive lymphocytes were decreased significantly in inactive versus active AOSD. Furthermore, CD32-positive monocytes correlated negatively with the levels of known disease activity markers and IL-23. These results suggest that CD32-expressing monocytes were decreased in active AOSD, although CD32-expressing leukocytes were increased in AOSD patients because CD32 has three subtypes and they have different functions in inflammation [26].

CD11c shares 63% amino acid homology with CD11b and also recognizes CD54 (also known as intercellular adhesion molecule 1) [32]. The integrin CD11c/CD18 has limited expression on the surface of macrophages or monocytes and dendritic cells, and was found to be less susceptible to activation than other β2-integrins [33]. A recent study evaluated several markers, including CD11a/b/c and CD64 (FcγRIA), on blood neutrophils and suggested that they could serve as useful biomarkers of sepsis and non-infectious systemic inflammatory response syndrome (SIRS) [34]. They found that increased blood neutrophils expressing CD11c could be a potential biomarker for sepsis and SIRS. In this study, there was no significant difference in the frequency of blood cells presenting CD11c between AOSD and HC. These data suggest that CD11c is not a good biomarker for the diagnosis or evaluation of disease activity in AOSD. However, these negative results suggest that CD11c cells could be used as a biomarker in the differential diagnosis between AOSD and sepsis [34].

CD206 (mannose receptor) is known as a C-type lectin, and is expressed by macrophages and dendritic cells. CD206 is a receptor for numerous ligands, including bacterial cell wall components and endogenous glycoproteins [35,36]. Functional polarization of macrophages can give rise to two different populations: classically activated macrophages (M1) and alternatively activated macrophages (M2). M2 macrophages are known to have high-level CD206 expression and a potent immunoregulatory capacity [37]. Monrad et al. [38] showed that lupus dendritic cells had diminished endocytic capacity, which correlated with decreased CD206 expression. However, one study in patients with COPD reported that overexpression of CD206 on lung alveolar macrophages was observed [39]. However, this study showed that the frequencies of CD206-positive monocytes were not different between patients with AOSD and HC. The frequencies of CD206 from granulocytes and lymphocytes were increased in patients with active versus inactive AOSD. There was no association between the CD206 cellular markers and disease activity markers, except for the correlation between CD206 granulocytes and IL-17. A recent study demonstrated an association between N2 polarization of neutrophils, like M2 macrophages, and their increased ability to undergo phagocytosis [40]. However, there are few studies about neutrophil-expressed CD206 and its function.

CD209 is also a trans-membrane C-type lectin, and can act in cell-adhesion and as a PRR for a wide range of microorganisms [41]. This PRR has been suggested to be linked to some immune diseases. One study evaluated the level of CD209 in psoriasis vulgaris lesions, and reported that CD209 was higher in psoriasis lesions compared with normal tissues [16]. A recent study showed the expression of CD209 on podocytes and its possible role in immune and inflammatory responses in lupus nephritis [42]. In this study, CD209 circulating granulocytes correlated with systemic scores, ESR, and levels of CRP, ferritin, LDH, IL-23, and IL-18. Also, the frequencies of CD209 from whole blood cells, granulocytes, monocytes, and lymphocytes were lower in inactive AOSD after treatment compared to active AOSD. It is not clear why CD209-expressing granulocytes showed positive correlations with known disease activity markers, although CD209-expressing granulocytes were not different between active AOSD patients and HCs.

Dectin-1 is a PRR of C-type lectins that is primarily expressed on myeloid cells and recognizes fungal cell wall components [43]. Also, dectin-1 triggers induction of proinflammatory cytokines, including IL-1β and TNF-α [44]. Monocytes from SLE patients showed low expression of dectin-1 compared with HC, and an inverse correlation between percent of dectin-1 cells and the disease activity score was found [17]. In this study, there was no significant difference in the frequency of blood cells presenting dectin-1 between AOSD and HC. Furthermore, the frequencies of circulating dectin-1-positive cells were not different between active and inactive phases of AOSD except for granulocytes presenting dectin-1. These data suggest that dectin-1 is not a good biomarker for the diagnosis or evaluation of disease activity in AOSD.

This study had several limitations. Several cellular markers were not compared with other febrile disorders in terms of their diagnostic value, and the sample sizes were too small in subgroup analyses according to the “monocyclic/polycyclic/chronic” classification and comparisons of follow-up samples. Further studies involving larger sample size are required for evaluating the usefulness of these markers in AOSD patients with control groups of other febrile disorders such as sepsis. However, we observed several PRR markers in AOSD, and evaluated their roles in evaluating disease activity in AOSD. Finally, we found a correlation between CD11b and CD32 and among several C-type lectins in AOSD patients.

## 4. Materials and Methods

### 4.1. Subjects

In total, 13 untreated AOSD patients, 19 RA patients (as a disease control), and 19 HC were included. Patients with AOSD were diagnosed according to Yamaguchi’s criteria after infections, malignancy, and other autoimmune diseases were excluded [45]. Almost all AOSD patients had arthritis (84.6%), therefore, we recruited RA patients as a disease control. RA patients satisfied the American College of Rheumatology (ACR) 1987 revised criteria for the classification of RA [46]. The HC were healthy individuals with no history of autoimmune, rheumatic, or other disease. Follow-up samples were collected from five patients after resolution of disease activity. Peripheral blood mononuclear cells (PBMCs) and serum were isolated from patients and controls.

The medical histories and clinical characteristics, including those identified during a physical examination, of all subjects were collected via a review of the subjects’ medical records and an interview with the subjects when the samples were collected. Laboratory findings, including a complete blood count, erythrocyte sedimentation rate (ESR), C-reactive protein (CRP), ferritin (normal level: 13–150 ng/mL for females and 30–400 ng/mL for males), and liver function tests were reviewed. The disease activity of AOSD was assessed according to the systemic disease score method, which is widely accepted and used [3]. Systemic disease scores range from 0 to 12, with 1 point for each of the following manifestations: fever, typical rash, pleuritis, pneumonia, pericarditis, hepatomegaly or abnormal liver function tests, splenomegaly, lymphadenopathy, leukocytosis (≥15,000/mm^2^), sore throat, myalgia, and abdominal pain. Inactive disease was defined as the patient no longer having systemic symptoms, such as fever, pleuritis, pericarditis, and pneumonia.

This study was approved by the Institutional Review Board of Ajou University Hospital (IRB No. AJIRB-BMR-SMP-13-380, 11 March 2014). Informed consent was obtained from all subjects.

### 4.2. Flow Cytometry of Surface-Stained Cells Presenting CD11b, CD11c, CD32, CD206, CD209, and Dectin-1

PBMCs were treated with ACK solution to lyse red blood cells (RBCs) and washed with phosphate-buffered saline (PBS), after which 1 × 10^6^ cells in each tube were incubated with allophycocyanin (APC)-labeled anti-CD4, fluorescein isothiocyanate (FITC)-labeled anti-CD11b, phycoerythrin–Cy7 (PE-Cy7)-labeled anti-CD11c, APC-labeled anti-CD32, PE-labeled anti-CD209, Peridinin chlorophyll protein (PerCP)-labeled anti-dectin-1 (eBioscience), FITC-labeled anti-CD8 (BD Biosciences, San Jose, CA, USA), and PE-Cy5-labeled anti-CD206 (BD PharMingen, San Diego, CA, USA), for 30 min at 4 °C. The same color-labeled antibodies were applied separately in different tubes. The stained cells were then washed with PBS and analyzed using a flow cytometer (FACSAria III; Becton Dickinson, San Jose, CA, USA) with 10,000 cells. The fluorescence-activated cell sorter (FACS) data were based on the gating of whole cells, granulocytes, monocytes, and lymphocytes; then, the specific markers were used to analyze the gated populations.

### 4.3. Cytokine Assay

Venous blood was extracted in test tubes containing an anticoagulant, EDTA. Aliquots of the peripheral blood were centrifuged (3500 rpm, 5 min, room temperature). After aspirating the supernatant, the samples were stored at −80 °C until required. Serum IL-17 levels were measured using a commercial enzyme-linked immunosorbent assay (ELISA) kit (R&D Systems, Minneapolis, MN, USA) according to the manufacturer’s protocol. Also, serum IL-18 and IL-23 were measured using commercial ELISA kits (eBioscience, San Diego, CA, USA) according to the manufacturer’s protocol.

### 4.4. Statistical Analyses

Data are shown as means ± standard deviation. Differences in frequencies of several cellular markers were determined using the Mann–Whitney *U*-test. Correlations between levels and disease activity markers were evaluated with Spearman’s correlation test. Statistical analyses were performed using SPSS for Windows software (ver. 23.0; IBM Corp., Armonk, NY, USA). A *p*-value < 0.05 was considered to indicate statistical significance.

## 5. Conclusions

Elevated frequencies of circulating CD11b-positive and CD32-positive cells and their correlations with disease activity markers and proinflammatory cytokines suggest that circulating CD11b-positive and CD32-positive cells contribute to the pathogenesis of AOSD.

## Figures and Tables

**Table 1:** Clinical characteristics of patients.

Clinical Features and Laboratory Results	AOSD Patients (*n* = 13)	RA Patients (*n* = 19)	HC (*n* = 19)
Age (years)	51.1 ± 20.5	48.2 ± 11.6	39.3 ± 14.7
Gender (F/M)	11/2	16/3	14/5
Fever	12 (92.3)		
Sore throat	9 (69.2)		
Skin rash	9 (69.2)		
Lymphadenopathy	4 (30.8)		
Splenomegaly	2 (15.4)		
Hepatomegaly	2 (15.4)		
Pericarditis	0 (0)		
Arthritis	11 (84.6)		
Hemoglobin, g/dL	11.3 ± 2.1	12.7 ± 1.5	
Leukocytes, μL	12,607 ± 4943	6084 ± 1908	
Platelets, ×10^3^/μL	311.2 ± 96.4	227.5 ± 56.4	
Ferritin, ng/mL	5282.5 ± 9707.7		
LDH, U/L	395.1 ± 231.6		
ESR, mm/h	61 ± 23.4	12.5 ± 10.9	
CRP, mg/dL	11.17 ± 6.99	0.29 ± 0.61	
AST/ALT, mg/dL	69 ± 57.4	26.1 ± 8.4	
AST, mg/dL	72.2 ± 87.6	24.5 ± 19.8	
Bilirubin, mg/dL	0.84 ± 0.55	0.75 ±0.21	
Albumin, g/dL	4.15 ± 0.54	4.4 ± 0.18	
ANA positivity	3 (23.1)	4 (21.1)	
RF positivity	1 (7.7)	14 (73.7)	
Systemic score	4.92 ± 1.85		
DAS-28		2.8 ± 1.25	

AOSD, adult-onset Still’s disease; RA, rheumatoid arthritis; HC, healthy controls; LDH, lactate dehydrogenase; ESR, erythrocyte sedimentation rate; CRP, C-reactive protein; AST, aspartate transaminase; ALT, alanine transaminase; ANA, antinuclear antibody; RF, rheumatoid factor. DAS-28, disease activity score including 28 joints. All values are presented as numbers (with percentages) or as means ± standard deviation. The systemic scoring system of Pouchot et al. [3] assigns a score from 0 to 12 with 1 point for each of the following manifestations: fever, typical rash, pleuritis, pneumonia, pericarditis, hepatomegaly or abnormal liver function test data, splenomegaly, lymphadenopathy, leukocytosis (≥15,000/mm^2^), sore throat, myalgia, and abdominal pain.

**Table 2:** Correlations between the frequencies of stained cells presenting several markers and disease activity markers in patients with adult-onset Still’s disease.

Disease Activity Marker	Correlation Coefficient, *r* (*p*-Value)						
	CD11b Whole Cells	CD11c Lymphocytes	CD32 Monocytes	CD209 Whole Cells	CD209 Granulocytes	CD209 Monocytes	Dectin-1 Lymphocytes
Systemic score	0.487 (0.041)	0.442 (0.066)	−0.559 (0.016)	0.486 (0.041)	0.484 (0.021)	0.424 (0.04)	0.363 (0.137)
Leukocytes	0.074 (0.769)	0.127 (0.616)	−0.393 (0.107)	0.283 (0.255)	0.212 (0.2)	0.413 (0.044)	0.089 (0.4)
Hemoglobin	−0.178 (0.481)	−0.376 (0.124)	0.309 (0.212)	−0.172 (0.496)	−0.274 (0.136)	−0.259 (0.15)	−0.178 (0.301)
Platelets	−0.022 (0.932)	0.122 (0.63)	−0.034 (0.893)	0.024 (0.925)	0.038 (0.44)	0.424 (0.04)	−0.182 (0.296)
ESR	0.285 (0.251)	0.541 (0.02)	−0.363 (0.139)	0.417 (0.085)	0.452 (0.03)	0.433 (0.036)	0.142 (0.339)
CRP	0.287 (0.248)	0.409 (0.092)	−0.468 (0.05)	0.395 (0.105)	0.428 (0.038)	0.298 (0.115)	0.319 (0.17)
Ferritin	0.286 (0.25)	0.672 (0.002)	−0.583 (0.011)	0.688 (0.002)	0.705 (0.001)	0.749 (<0.001)	0.829 (0.001)
LDH	0.58 (0.012)	0.539 (0.021)	−0.412 (0.089)	0.449 (0.061)	0.535 (0.011)	0.55 (0.009)	0.694 (0.009)
Bilirubin	0.028 (0.911)	−0.01 (0.967)	0.493 (0.038)	0.061 (0.809)	0.045 (0.43)	−0.017 (0.473)	0.199 (0.279)
AST	0.635 (0.005)	0.25 (0.317)	−0.276 (0.268)	0.247 (0.324)	0.331 (0.09)	0.394 (0.053)	0.438 (0.089)
ALT	0.335 (0.174)	−0.045 (0.858)	0.127 (0.616)	−0.119 (0.639)	−0.072 (0.388)	0.052 (0.42)	0.046 (0.447)
IL-17	0.106 (0.674)	0.229 (0.227)	−0.189 (0.452)	0.326 (0.187)	0.359 (0.072)	0.321 (0.097)	0.491 (0.063)
IL-18	0.564 (0.015)	0.452 (0.06)	−0.358 (0.145)	0.441 (0.067)	0.411 (0.045)	0.159 (0.265)	0.525 (0.049)
IL-23	0.529 (0.024)	0.367 (0.135)	−0.476 (0.046)	0.386 (0.113)	0.458 (0.028)	0.199 (0.214)	0.255 (0.224)

ESR, erythrocyte sedimentation rate; CRP, C-reactive protein; LDH, lactate dehydrogenase; AST, aspartate transaminase; ALT, alanine transaminase. Spearman’s correlation coefficients were calculated. The systemic scoring system of Pouchot et al. [3] assigns a score from 0 to 12 with 1 point for each of the following manifestations: fever, typical rash, pleuritis, pneumonia, pericarditis, hepatomegaly or abnormal liver function test data, splenomegaly, lymphadenopathy, leukocytosis (≥15,000/mm^2^), sore throat, myalgia, and abdominal pain.

**Table 3:** Correlations between each cell surface marker in patients with adult-onset Still’s disease.

	Correlation Coefficient, *r* (*p*-Value)				
	CD11c	CD32	CD206	CD209	Dectin-1
CD11b	0.243 (0.166)	0.853 (<0.001)	0.108 (0.334)	0.006 (0.49)	0.147 (0.324)
CD11c		0.263 (0.146)	0.765 (<0.001)	0.317 (0.1)	0.811 (0.001)
CD32			0.108 (0.334)	−0.231 (0.178)	0.035 (0.457)
CD206				0.624 (0.003)	0.846 (<0.001)
CD209					0.615 (0.017)
